# Phase Stability and Site Preference of Tb-Fe-Co-V Compounds

**DOI:** 10.1155/2013/919182

**Published:** 2013-06-26

**Authors:** Jing Sun, Jiang Shen, Ping Qian

**Affiliations:** Institute of Applied Physics, University of Science and Technology, Beijing 100083, China

## Abstract

The effect of cobalt on the structural properties of intermetallic Tb_3_Fe_27.4−*x*_Co_*x*_V_1.6_ with Nd_3_(Fe,Ti)_29_ structure has been studied by using interatomic pair potentials obtained through the lattice inversion method. Calculated results show that the preferential occupation site of the V atom is found to be the 4i(Fe3) site, and Fe atoms are substituted for Co atoms with a strong preference for the 8j(Fe8) site. The calculated lattice constants coincide quite well with experimental values. The calculated crystal structure can recover after either an overall wide-range macrodeformation or atomic random motion, demonstrating that this system has the stable structure of Nd_3_(Fe,Ti)_29_. All these prove the effectiveness of interatomic pair potentials obtained through the lattice inversion method in the description of rare-earth materials.

## 1. Introduction

In 1993, it had been first found that the structure of the compound Nd_2_Fe_19−*x*_Ti_*x*_ suggested to be a Nd_3_(Fe,Ti)_29_-type structure with monoclinic symmetry [1]. After lots of investigation and disputation [2–4], it indicates that the Space Group (S.G.) of Nd_3_(Fe,Ti)_29_-type structure belongs to A2/m rather than P2_1_/c.

In the A2/m space group description, the lattice cell with two R_3_Fe_29_ formulas consists of 64 atoms. The rare-earth ions occupy two nonequivalent crystallographic sites (2a and 4i), and the iron atoms occupy 11 sites (one 2c, one 4g, one 4e, four 4i and four 8j). The crystallographic data for Nd_3_Fe_27.5_Ti_1.5_ compound (Space Group no. 12 (A2/m), A12/m1, unique axis b, cell choice 2) is on page 161 of [5]. The 3 : 29 compounds are deemed to consist of tetragonal ThMn_12_-type (1 : 12) and rhombohedral Th_2_Ni_17_-type (2 : 17) segments in a ratio of 1 : 1 [6]. In fact, the binary compounds R_3_Fe_29_ are metastable, which can be seen as the intrinsic prototype of R_3_(Fe,T)_29_, since R_3_(Fe,T)_29_ can be made stable with a moderate amount of a ternary element T (T = V, Ti, Cr, or Mo, etc.). From that time on, the ternary intermetallic compounds R_3_(Fe,T)_29_ (R = a rare-earth element or Y, and T = a stabilizing element) have attracted attention, as well as many quaternary compounds [7–14]. 

As well known, the substitution of cobalt for iron in rare-earth-transition metal intermetallics has a remarkable effect on their magnetic properties. Comparing with iron, cobalt has a different electronic structure and therefore lead to a different local anisotropy. Hence, the structure and the magnetic properties of 3 : 29 compounds are strongly affected by the substitution of iron by cobalt. However, when more than 40% of substitution of iron by cobalt atoms occurs, a large number of stabilizing atoms are demanded. If not, a disordered modification of hexagonal Th_2_Ni_17_-type structure will be formed [10, 11, 13, 14]. 

So far, there are some investigations on the Tb-Fe-Co-V and Tb-Fe-Co-Cr systems [9, 11–14]. For Tb_3_Fe_29−*x*_V_*x*_ compounds, in the temperature range between 5 K and 200 K, a FOMP occurs [15]. The spin reorientation has been observed in the Tb-Fe-Co-V system, and the Curie temperature and saturation magnetisation decrease with the increase of Co content [13].

In this paper, our investigation focuses on the phase stability and site preference of Co in Tb_3_Fe_27.4−*x*_Co_*x*_V_1.6_ (*x* = 0.0, 0.1, 0.2, 0.3 and 0.4) compounds. The interatomic pair potential are obtained by Chen's lattice inversion method, whose theory is exhibited in the second part. The comparison of the calculated results with the experimental data is shown in Section 3. And the fourth part is the conclusion and discussion.

## 2. Methodology

Chen et al. proposed a concise inverse method [16–21] based on the modified Möbius inversion in number theory, which can be applied to obtain the photon density of states [16], to solve the inverse blackbody radiation problem for remote sensing [16], to unify the Debye and Einstein approximations in a general mathematics system [20], and to extract the interatomic pair potentials from ab initio calculated cohesive energy curves in pure metals and intermetallic compounds [17–19, 22, 23], metal/ceramic interface [24], and metal/oxide interface [25], as well as in carbides with complex structures [26–28], with high convergence speed. 

Here, we take a single element crystal as an example to explain how to use Chen's lattice inversion theorem to obtain the interatomic pair potential from the first-principle cohesive energy curve. Suppose that the crystal cohesive energy can be expressed as the sum of interatomic pair potentials

(1) $$\begin{array}{c} E\left(x\right)=\frac{1}{2}\sum_{r_{i}\neq 0}\varphi \left(\vec{r_{i}}\right)=\frac{1}{2}\sum_{n=1}^{\infty }r_{0}\left(n\right)\varphi \left[b_{0}\left(n\right)x\right], \end{array}$$

where *x* is the nearest neighbor distance, $\vec{r_{i}}$ is the position vector of the *i*th atom in the lattice, *b*_0_(*n*)*x* is the *n*th nearest neighbour distance with *b*_0_(1) = 1, and *r*_0_(*n*) is the *n*th coordination number. Obviously, {*b*_0_} is a totally ordered set but, in most cases, not a multiplicative semigroup. In this case, we need to extend it to a totally ordered multiplicative semigroup {*b*} with *b*(1) = *b*_0_(1) = 1. Namely, for any *b*(*j*), *b*(*k*)∈{*b*}, we have *b*(*j*)*b*(*k*)∈{*b*}. Now *E*(*x*) can be rewritten as

(2) $$\begin{array}{c} E\left(x\right)=\frac{1}{2}\sum_{n=1}^{\infty }r\left(n\right)\varphi \left[b\left(n\right)x\right], \end{array}$$

where the extended coordination number *r*(*n*) satisfies the following rule:

(3) $$\begin{array}{c} r\left(n\right)=\{\begin{array}{cc} r_{0}\left(b_{0}^{-1}\left[b\left(n\right)\right]\right), & b\left(n\right)\in \left\{b_{0}\right\},\\ 0, & b\left(n\right)\notin \left\{b_{0}\right\}. \end{array} \end{array}$$

It is clear that {*r*(*n*)} is uniquely determined by crystal geometrical structure. Now we can extract pair potential *φ* from (2) as

(4) $$\begin{array}{c} \varphi \left(x\right)=2\sum_{n=1}^{\infty }I\left(n\right)E\left[b\left(n\right)x\right], \end{array}$$

where *I*(*n*), *n* = 1, 2,…, *∞*, and any *b*(*n*) satisfying *b*(*n*) | *b*(*t*), {*I*(*n*)} can be determined by

(5) ∑b(n) ∣ b(t)I(b−1[b(n)])r(b−1[b(t)b(n)])=δt,1              (t=1,2,…,∞),

where *δ* is the Kronecker delta. It is easy to prove that (5) has and only has a single solution set {*I*(*n*)}, which, like *r*{(*n*)}, is uniquely determined by crystal geometrical structure, not related to element category.

This means that we can take advantage of number theory to extract the interatomic pair potentials form cohesive energy curve(s). At this stage, modified Möbius inversion can be adopted to extract the pair potential curve data and then fit these data on the basis of Morse function [29] to determine the potential parameters. Consider

(6) $$\begin{array}{c} \varphi \left(x\right)=D_{0}\left(e^{-\gamma (x/R_{0}-1)}-2e^{-(\gamma /2)(x/R_{0}-1)}\right), \end{array}$$

where *x* indicates the distance between two atoms and *R*_0_ is the equilibrium distance, with *D*_0_ and *γ* being parameters without unit.

**Figure 1:**
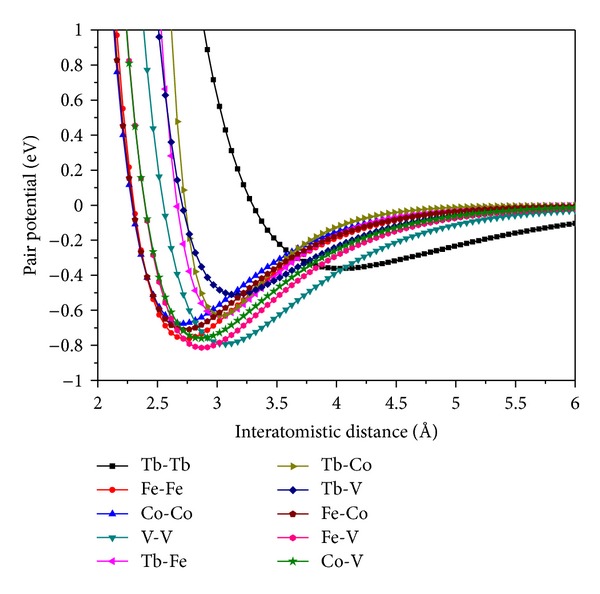
Potentials in the system of Tb-Fe-Co-V.

For the readers' convenience, several important potential parameters are shown in Figure 1.

## 3. Calculated Results

In this paper, energy minimization is carried out using a conjugate gradient method. The cut-off radius is 14 Å. In order to reduce statistical fluctuation, we take the super-cell containing 1280 atoms, (Tb_3_Fe_27.4−*x*_Co_*x*_V_1.6_)_40_, for simulation. 

There are no reports in the literature on the existence of the binary structure Tb_3_Fe_29_, which can be seen as the prototype of R_3_(Fe,T)_29_. In the calculation procedure, the initial lattice constants of Tb_3_Fe_29_ are randomly chosen in a certain range. Under the control of the interatomic pair potentials, the energy minimization is carried out. After repeated relaxation, the space group maintains A2/m, the atomic site occupation is similar to that of Nd_3_(Fe,Ti)_29_, and the structure will be stabilized with lattice constants *a* = 10.525 Å, *b* = 8.433 Å, *c* = 9.696 Å, and *β* = 96.91° (Table 1). The randomness of the initial structure in a certain range and the stability of the final structure illustrate that Tb_3_Fe_29_ has the topological invariability with respect to the existing Nd_3_(Fe,Ti)_29_ structure. Hence, it furnishes convincing evidence that the interatomic pair potentials are reliable for the study of structural material characteristics.

**Figure 2:**
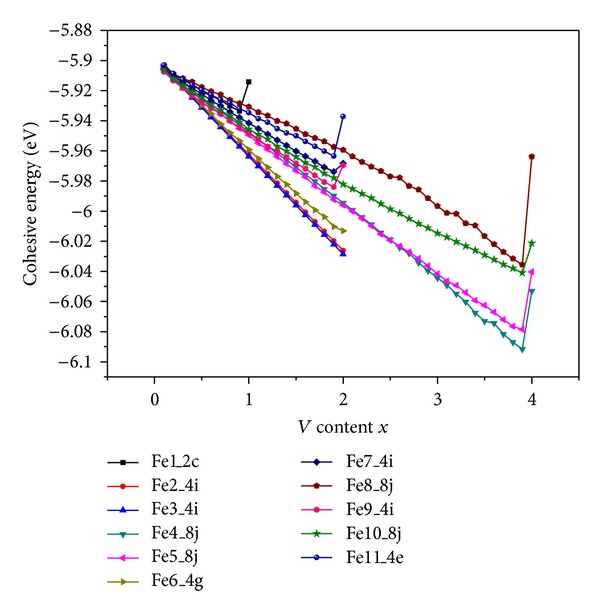
Calculated cohesive energy of Tb_3_Fe_29−*x*_V_*x*_ as a function of the V content *x*.

Substitute the atoms of ternary element vanadium for the randomly chosen iron atoms at a certain lattice site, and then make the lattice relaxation. In this calculation procedure, energy minimization is taken as a criterion of the stability. The cohesive energy of Tb_3_Fe_29−*x*_V_*x*_ on the content of ternary addition is evaluated and illustrated in Figure 2. The values of energy in the figure are statistically averaged over the calculations of 20 samples. Figure 2 shows that the cohesive energy is lower when V atoms are substituted for Fe atoms at the 4i(Fe3) site than the other sites, so V should prefer the 4i(Fe3) site for *x* ≤ 2.0. The calculated lattice constants of compound Tb_3_Fe_27.4_V_1.6_ are compared with the experimental values [13], which are shown in Table 2. Substitute the V atoms for a randomly selected part of Fe atoms at the 4i(Fe3) sites, thus forming the (Tb_3_Fe_27.4_V_1.6_)_40_. Then make use of the conjugate gradient method to minimize the system energy, as an approximation of a practical relaxation process. Furthermore, we take the (Tb_3_Fe_27.4_V_1.6_)_40_ structure with each atom random shifted 0.6 Å from their equilibrium position to test the structural stability. Each atom in a disturbed cell can recover its equilibrium position under the interaction of interatomic pair potentials. The results show that the lattice constants are in good agreement with the experimental data [13], still retaining *a* = 10.5433 Å, *b* = 8.4647 Å, *c* = 9.7196 Å, and *β* = 97.0681° (Table 3). Thus, the stability of the lattice and the effectiveness of the interatomic pair potentials are verified.

**Figure 3:**
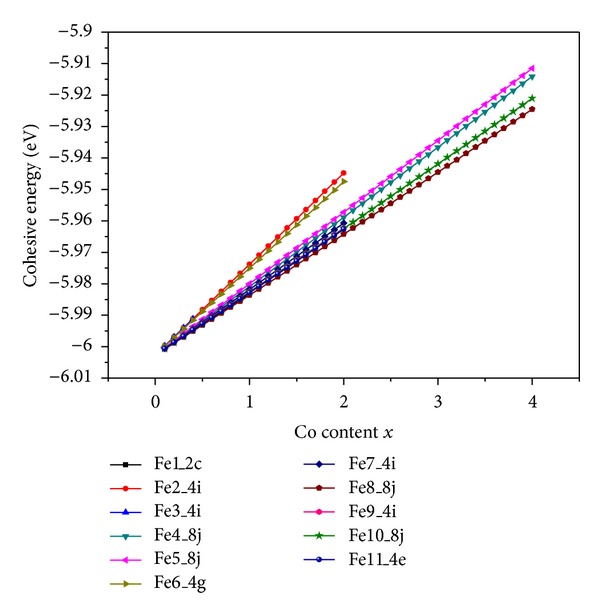
Calculated cohesive energy of Tb_3_Fe_27.4−*x*_Co_*x*_V_1.6_ as a function of the Co content *x*.

In Tb_3_Fe_27.4−*x*_Co_*x*_V_1.6_ compounds, substitute the atoms of quaternary element cobalt for the randomly chosen iron atoms at a certain lattice site, and then make the lattice relaxation. Based on the ternary compounds Tb_3_Fe_27.4_V_1.6_ and the site occupation of cobalt in quaternary system Tb_3_Fe_27.4−*x*_Co_*x*_V_1.6_ has been investigated. The dependences of the cohesive energy of Tb_3_Fe_27.4−*x*_Co_*x*_V_1.6_ on the content of quaternary addition are evaluated and illustrated in Figure 3. It is shown that the calculated cohesive energy increases least significantly while cobalt atoms occupy 8j(Fe8) site. Therefore, Fe atoms are substituted for Co atoms with a strong preference for the 8j(Fe8) site. The calculated lattice constants are compared with the experimental values [13], which are shown in Table 2. It states clearly that the lattice parameters *a*, *b* (but not *c*) of the compounds are decreased with increasing cobalt concentration due to the fact that the iron atoms are replaced by the smaller cobalt atoms. 

The stability of the calculated structure is further checked through molecular dynamics. The cell constants are traced to higher temperatures, as shown in Table 4. Using MD (molecular dynamics) NPT ensemble, with *P* = 1 atm, *t* = 0.001 ps, dynamic simulations for Tb_3_Fe_27.4−*x*_Co_*x*_V_1.6_ are carried out at temperatures of 300, 500, and 700 K. The lattice constants change very little with respect to temperature variation, thus the structural stability is again verified. The comparisons of potential energy and kinetic energy at different temperatures are shown in Table 5. It can be seen from the table that both the values of potential energy and kinetic energy increase with the increasing temperature, but the contribution of interatomic potentials to the internal energy of the system is much larger than that from thermal motion energy. Therefore, the crystal structure at different temperature is basically determined by the interatomic pair potentials.

## 4. Conclusions

In this paper, we have made an investigation of the phase stability and effect of cobalt atoms element on the structural properties of Tb_3_Fe_27.4−*x*_Co_*x*_V_1.6_ crystals. The energies of the Tb-Fe-Co-V systems are calculated from these effective interatomic pair potentials. The calculation results show that vanadium atoms preferentially occupy 4i(Fe3) site, and cobalt prefer the 8j(Fe8) site. Besides, due to the fact that iron atoms are replaced by the smaller cobalt atoms, the lattice parameters *a*, *b* (but not *c*) of the compounds decreased with increased concentration.

Above all, with the interatomic potentials from lattice inversion, the structural properties have been well reproduced and are in agreement with the experimental data. This suggests that the potentials are successfully used to calculate the structural properties of rare-earth compounds Tb_3_Fe_27.4−*x*_Co_*x*_V_1.6_, which is important for future work on material structure research.

## Figures and Tables

**Table 1:** Final crystal lattice constants of Tb_3_Fe_29_ corresponding to random initial structures.

Initial state		Final state	
*a*, *b*, *c* (Å)	*α*, *β*, *γ* (deg)	*a*, *b*, *c* (Å)	*α*, *β*, *γ* (deg)
10, 8, 9	90, 95, 90	10.525, 8.433, 9.696	90, 96.91, 90
11, 10, 10	90, 100, 90	10.525, 8.433, 9.696	90, 96.91, 90
10, 10, 10	90, 90, 90	10.525, 8.433, 9.696	90, 96.91, 90
13, 10, 10	90, 95, 90	10.525, 8.433, 9.696	90, 96.91, 90
12, 12, 12	90, 90, 90	10.525, 8.433, 9.696	90, 96.91, 90
10, 10, 10	85, 90, 88	10.525, 8.433, 9.696	90, 96.91, 90
8, 8, 8	90, 90, 90	10.525, 8.433, 9.696	90, 96.91, 90

**Table 2:** Comparison of calculated and experimental unit cell parameters.

	*x* = 0	*x* = 0.1	*x* = 0.2	*x* = 0.3	*x* = 0.4
	Tb_3_Fe_27.4_V_1.6_	Tb_3_Fe_27.3_Co_0.1_V_1.6_	Tb_3_Fe_27.2_Co_0.2_V_1.6_	Tb_3_Fe_27.1_Co_0.3_V_1.6_	Tb_3_Fe_27.0_Co_0.4_V_1.6_
*a* (Å)					
Cal.	10.5433	10.5424	10.5418	10.5411	10.5406
Exp.	10.557 (1)	10.545 (1)	10.537 (1)	10.519 (1)	10.511 (1)
Err. (%)	−0.1298	−0.0247	0.2168	0.2101	0.2816
*b* (Å)					
Cal.	8.4647	8.4647	8.4643	8.4638	8.4635
Exp.	8.497 (1)	8.496 (1)	8.490 (1)	8.476 (1)	8.461 (1)
Err. (%)	−0.3801	−0.3684	−0.3027	−0.1439	0.0295
*c* (Å)					
Cal.	9.7196	9.7189	9.7185	9.7181	9.7176
Exp.	9.669 (1)	9.672 (1)	9.669 (1)	9.658 (1)	9.645 (1)
Err. (%)	0.5233	0.4849	0.5119	0.6223	0.7527
*β* (deg)					
Cal.	97.0681	97.0703	97.0698	97.0690	97.0691
Exp.	96.89	96.72	96.72	96.71	96.74
Err. (%)	0.18	0.31	0.3617	0.3712	0.3402

**Table 3:** Comparison of related lattice constants before and after atomic random motion of 0.6 Å for Tb_3_Fe_27.4_V_1.6_.

	*a* (Å)	*b* (Å)	*c* (Å)	*β* (deg)
Before random motion	10.5433	8.4647	9.7196	97.0681
After random motion of 0.6 Å	10.5433	8.4647	9.7196	97.0681
Experimental [13]	10.557 (1)	8.497 (1)	9.669 (1)	96.89

**Table 4:** The cell constants of Tb_3_Fe_27.4−*x*_Co_*x*_V_1.6_ under different temperatures.

Compounds	Temperature	*a* (Å)	*b* (Å)	*c* (Å)	*β* (deg)
*x* = 0.0	300 K	10.5269	8.4339	9.6954	96.8980
	500 K	10.5229	8.4309	9.6962	96.9095
	700 K	10.5250	8.4360	9.6958	96.9115

*x* = 0.1	300 K	10.5240	8.4329	9.6952	96.9190
	500 K	10.5241	8.4317	9.6952	96.8885
	700 K	10.5252	8.4320	9.6950	96.9185

*x* = 0.2	300 K	10.5249	8.4310	9.6954	96.9180
	500 K	10.5241	8.4309	9.6954	96.9025
	700 K	10.5220	8.4330	9.6962	96.9195

*x* = 0.3	300 K	10.5250	8.4331	9.6959	96.9195
	500 K	10.5280	8.4328	9.6962	96.9005
	700 K	10.5282	8.4331	9.6950	96.9095

*x* = 0.4	300 K	10.5252	8.4338	9.6962	96.9000
	500 K	10.5252	8.4313	9.6960	96.8990
	700 K	10.5229	8.4330	9.6956	96.9180

**Table 5:** The potential and kinetic energies of Tb_3_Fe_27.4−*x*_Co_*x*_V_1.6_ under different temperatures.

Compounds	Temperature	*E*_*p*_ (eV)	*E*_*k*_ (eV)	*E*_total_ (eV)	*E*_*p*_/*E*_total_
*x* = 0.0	300 K	−1907.8644	12.4699	−1895.3944	1.0066
	500 K	−1898.9049	20.1887	−1878.7162	1.0107
	700 K	−1891.3703	29.0479	−1862.3224	1.0156

*x* = 0.1	300 K	−1907.4917	12.7817	−1894.7100	1.0067
	500 K	−1898.5085	20.1824	−1878.3261	1.0107
	700 K	−1889.7135	27.8954	−1861.8181	1.0150

*x* = 0.2	300 K	−1907.2399	13.0351	−1894.2048	1.0069
	500 K	−1898.6019	20.8418	−1877.7601	1.0111
	700 K	−1890.0060	28.8429	−1861.1631	1.0155

*x* = 0.3	300 K	−1905.9974	12.4685	−1893.5289	1.0066
	500 K	−1897.0235	19.9308	−1877.0927	1.0106
	700 K	−1889.9966	29.3169	−1860.6797	1.0158

*x* = 0.4	300 K	−1905.6689	12.7901	−1892.8788	1.0068
	500 K	−1898.0230	21.6214	−1876.4016	1.0115
	700 K	−1889.3255	29.3861	−1859.9394	1.0158
